# Genius is Largely a Matter of Making a Continuous Effort: A Reappraisal of the Contributions of H. William Scott, Jr., MD, A Surgeon Nonpareil

**DOI:** 10.1097/AS9.0000000000000312

**Published:** 2023-07-27

**Authors:** Walter H. Merrill, Sunil K. Geevarghese

**Affiliations:** From the * Department of Cardiac Surgery, Vanderbilt University Medical Center, Medical Center North, Nashville, TN; † Section of Surgical Sciences, Vanderbilt University Medical Center, Medical Center North, Nashville, TN

There are some individuals who, perhaps through good fortune, circumstance, or the application of an indomitable will, seem to have an outsized influence on the growth and reputation of a particular institution. Such was the case at Vanderbilt University’s School of Medicine during the time of H. William Scott, Jr., MD. His remarkable story is one of meaningful institutional progress and personal contributions and is worthy of revisiting at this time.

## EARLY YEARS THROUGH MEDICAL SCHOOL

H. William Scott, Jr., was born on August 22, 1916, in Graham, North Carolina. It seems that he was a very accomplished student, as he started the 7th grade at the age of 10 years.

During his time in high school, he played sports and the trumpet and was elected president of his class. His troubles with a duodenal ulcer began when he was a high school student and would haunt him later in life. His initial plan was to study engineering, but, as a freshman in high school, he witnessed his cousin, Dr. Mel Thompson, reduce the dislocated shoulder of an injured football player on the field. This proved to be an inspiration to take up medicine instead. He would later say, “I just stuck with it and thought medicine was what I wanted to get into.”

After attending high school in Graham, North Carolina, which then only went through the 11th grade, he attended Darlington School, a boarding school in Rome, Georgia, for 1 year. He was subsequently named a Distinguished Alumnus of Darlington School in 1985.

He attended the University of North Carolina (UNC), where he was inducted into Phi Beta Kappa and graduated in 1937. He once said that he was very confident regarding his qualifications for medical school, for he applied only to Harvard and was admitted. He would later be named Distinguished Alumnus of UNC as well.

Dr. Scott graduated from Harvard Medical School in 1941 and was inducted into Alpha Omega Alpha. In his first year at Harvard, he became interested in the anatomy of the nervous system and later in neurology. He subsequently decided to pursue neurosurgery because he became energized about the prospect of doing something about the lesions rather than just diagnosing them.

## RESIDENCY TRAINING IN BOSTON AND BALTIMORE

Following medical school, he undertook postgraduate training, interning first in pathology and later in surgery at Peter Brent Brigham Hospital and Boston Children’s Hospital under Drs. William Ladd and Robert Gross. He also was the Harvey Cushing Fellow in Neurosurgery at Boston Children’s and Peter Brent Brigham. His active duodenal ulcer kept him out of the service during World War II. During the war years, he married Mary Louisa Vanamee on October 17, 1942, and they subsequently had 4 children: H. William Scott, III, Mary Elizabeth Scott (McWilliams), Virginia Wright Scott (Mireya Lovelight VanAmee), and Patricia Vanamee Scott (Staley).

Dr. Robert Gross subsequently paid him an unusual compliment in his textbook on pediatric surgery, The Surgery of Infancy and Childhood: Its Principles and Techniques. Although Scott was not named, there is substantial evidence that he is the person referred to in Chapter 2 as Gross’ “…chief resident whose training was seemingly inadequate for this post but who was available because an active duodenal ulcer kept him from military duty.” Gross goes on to describe this person as possessing “…a real fondness and affection for children; he was indefatigable in his efforts to make them comfortable and happy; he was almost constantly at the bedside of those who were desperately ill.”

Some years later, Scott wrote to Dr. Gross, “You served as a role model for all of the members of the house staff of those days and you set us a standard that was awfully hard to live up to.” (Box 3, Folder 35: Letter H. William Scott, Jr., MD, to Robert Gross, MD, June 30, 1980. Personal Communication). He would also compliment the house staff members of the 2 institutions when he wrote, “All of you (residents at Boston Children’s Hospital and Brigham) were such superb hard driving interesting people that I very early decided that I wanted to cast my lot with the type of people that were represented in surgery.” (Box 8, Folder 3: Letter H. William Scott, Jr., MD, to Robert Zollinger, MD, June 10, 1985. Personal Communication.).

As he finished his training in Boston, he felt that he needed more experience in the care of adult patients. At that point, he decided to leave Boston and to undertake additional training with Dr. Alfred Blalock in Baltimore. From 1946 to 1947 he was at Johns Hopkins Hospital under Dr. Blalock, first as Surgery Assistant Resident from January 1946 to October 1946, and then as Surgical Resident, from October 1946 to May 1947.

He subsequently said in an interview that was published in the 1982 Canby Robinson Society bulletin, “I didn’t feel I had adequate adult surgical experience (after his residency in Boston). I then had two years with Alfred Blalock at Johns Hopkins. He was a stimulating teacher, splendid to work with, but also very demanding. He expected you to do everything to perfection; he would accept nothing less than an approximation of perfection. He had a wonderful trick of stimulating people to make them play above their heads…Studying surgery with Blalock is like a writer studying his art with Steinbeck or Dickens.”

## TIME ON THE FACULTY AT JOHNS HOPKINS

As he finished his time as a resident surgeon, Dr. Scott was then appointed as an instructor in surgery, after which he rose through the ranks to Associate Professor of Surgery in 1950. He would say that it was a very exciting time to be at Johns Hopkins, mainly due to the worldwide interest in Dr. Blalock and his ground-breaking achievements in congenital heart surgery. He commented regarding the working environment of the Hopkins Department of Surgery: “… When I got to Hopkins I found that under Dr. Blalock it was very easy to do laboratory work and in fact he pressed all of us to get into the laboratory while we were on clinical services and work nights, weekends, etc…Dr. Blalock stimulated me to get to work on projects by this rather negative attitude. ‘Oh, Bill, I think this has been done before and I really don’t think you can do it.’…I reacted by trying to show him that I could do it and that it was interesting and that it hadn’t been done before and that it was worthwhile. At Hopkins at that time, anyone who had a project he wanted to work on in the laboratory could do so. It was as simple as that. Once I got going in the lab at Hopkins I never wanted to get out of it, including the first 15 to 20 years at Vanderbilt.” (Box 3, Folder 35: Letter H. William Scott, Jr., MD, to Robert Gross, MD, September 19, 1988. Personal Communication.).

Scott was recognized for his work as he rapidly ascended the academic promotional ladder and began some initiatives in the laboratory. Gaining national recognition, he was considered a possible successor to Dr. Barney Brooks, Chair of the Department of Surgery at Vanderbilt. He had this to say about the opportunity, “Dr. Blalock emphasized to me that it was basically a fine institution that had fallen on hard times and needed developing, and he essentially told me that I could not turn down the opportunity and that he wanted me to go and do everything I could to turn Vanderbilt into a Hopkins.” (Box 3, Folder 35: Letter H. William Scott, Jr., MD, to Robert Gross, MD, September 19, 1988. Personal Communication.). Blalock knew the situation at Vanderbilt very well, as he had left Hopkins after 3 years of surgical training and had gone there as Brooks’ first Chief Resident in 1925. He subsequently remained on the faculty at Vanderbilt until he returned to Hopkins as the Chair of the Department of Surgery in 1941.

## MOVE TO VANDERBILT AND REORGANIZATION OF THE DEPARTMENT OF SURGERY AND TRAINING PROGRAM

On October 19, 1951, The Nashville Banner announced the appointment of Dr. Scott as the Professor of Surgery and Chairman of the Department of Surgery at Vanderbilt University. He was to take over the reins held by Dr. Barney Brooks, who had held these positions since 1925. He came to Vanderbilt on January 1, 1952, and, at that time, he was one of the youngest individuals ever appointed in the US to such a post. When he arrived, he found that there was no private clinic, nor did he find a postoperative recovery room, surgical intensive care unit, or emergency service. Moreover, there was a limited number of surgical house staff. It was his plan to create divisions within the surgical specialties and to develop residencies in urology, orthopedics, and neurosurgery. He encountered persistent acrimony that arose out of the town-gown problem. This had originated from the forced resignations of faculty that came as a result of the reorganization of the medical school in 1925 and was still bitterly remembered.

Scott began making changes within the Department of Surgery in his first year. Drs. Scott and Rollin Daniel, along with the surgical residents, painted the halls of the surgical wards on the weekends. This was a very noteworthy endeavor to many individuals, including Duncan Killen, who later wrote, “You were in your overalls with the House Officers painting what was then the Women’s Surgical Ward. I think that projected the tone of your desire and push to bring the Vanderbilt Surgical Program into a modern era. You taught those around you by example more than you realize.” (Box 43, Letters, H. William Scott, Jr., MD 1923–1997, Letter to H. William Scott, Jr. from Duncan Killen, MD, January 31, 1989. Personal Communication.).

The first open-heart operation at Vanderbilt was performed by Scott in 1954. He is acknowledged by many to be one of the very first surgeons to carry out an open-heart repair of the tetralogy of Fallot and of an aorta-pulmonary window. However, Dr. Scott continued to be troubled by his symptoms of peptic ulcer disease, and he underwent a vagotomy and antrectomy by Dr. L.W. Edwards and Dr. Rollin Daniel.

Shortly thereafter, a thoracic surgery residency led by Dr. Rollin Daniel was approved, and the emergency service and pediatric surgery services were organized. (Scott, HW, Jr. 1955-1956. In: Scott, Jr. HW, Herrington JL, Rosenfeld L, Sawyers JL, eds. History of Surgery at Vanderbilt. Nashville; 1996:107-110). By 1957, the integration of the various surgical residencies into a single 5-year program was accomplished and included surgery residents from Vanderbilt, Thayer General Veterans Affairs Hospital, and the Middle Tennessee Tuberculosis Hospital. The S.R. Light Laboratory for Surgical Research was a gift from the father of Rudolph Light, Chief Resident under Dr. Brooks in 1946–1947, and it was dedicated on April 20, 1955.

By July 1958, there were 12 interns, and teaching rounds were held 6 days per week. There was a 5-year residency that offered training in both general and thoracic surgery. Evidence of tremendous progress in research was demonstrated by the fact that there were 12 papers from Vanderbilt presented at the Surgical Forum of the American College of Surgeons.

In 1960, Dr. John Sawyers was named Chief of Surgical Service at Nashville General Hospital, and thus began a very fruitful period of affiliation between Vanderbilt and Nashville General Hospitals. By the academic year 1963, the Clinical Research Center had been enlarged to 21 beds, a pediatric surgery floor was developed, the Veterans Administration hospital in its new location had been completed, and the integration of surgical residencies from the Veterans Administration and the Nashville General Hospitals into Vanderbilt had been completed. Also, the first transplantation of a kidney into a patient was accomplished. Subsequently, the surgical residency would expand to include multiple rotations at St. Thomas Hospital, and there would be a pediatric surgical rotation at Children’s Mercy Hospital in Kansas City. (Scott, HW, Jr. 1960-1961. In: Scott, Jr. HW, Herrington JL, Rosenfeld L, Sawyers JL, eds. History of Surgery at Vanderbilt. Nashville; 1996:133-138).

Further progress and growth in the Department of Surgery culminated in an annual gathering of current and former residents and faculty, which was held in conjunction with the Barney Brooks Lecture. At the meeting held on March 18, 1972, in honor of his 20 years of service to Vanderbilt, the annual meeting was renamed the H. William Scott, Jr. Society. Dr. Scott always referred to it as the Resident’s Society.

At the first meeting of the H. William Scott, Jr. Society, Dr. Dawson Conerly, the most senior living former surgical resident and President of the group, said that Dr. Scott taught us that “…genius is largely a matter of making a continuous effort…And he taught us the value of discontent, that it can be the fire under the boiler of progress.”

The Section of Surgical Sciences was formed in 1975. Dr. Scott was named Director of the Section and Chairman of the Department of Surgery. He would describe the purpose of the formation of the section as follows: “…to maintain unity and cohesiveness of purpose and objectives among the surgical disciplines. What are now divisions of surgery will become departments in the Section of Surgical Sciences. Each of the present divisions will have equal status and autonomy. At the same time, the organization will provide an umbrella to keep us all together. We don’t want to weaken the interdisciplinary cooperation typical of Vanderbilt’s approach.”^1^ He would later add, “Any division which became highly productive with an approved residency and strength in its faculty representation should be sponsored by the Section for elevation to autonomous departmental status. All were linked by the administrative unity of the Section of Surgical Sciences.” (Box 8, Folder 1: Letter H. William Scott, Jr., MD, to Clayton Wheeler, Jr., MD, February 15, 1979. Personal Communication.). He firmly believed that problems that might occur should be left to the Section leadership, adding that “…problems which involve professional matters are decided upon by the Executive Committee (of the Section) and we believe more appropriately than by medical school or hospital administrators.” (Box 6, Folder 8: Summary note of H. William Scott, Jr., MD, to Roscoe Robinson, MD, undated. Personal Communication.).

Initially, Scott was highly interested in cardiovascular problems, but his interest in gastrointestinal problems and other illnesses seemed to be ignited by his own operation for peptic ulcer disease. He became a highly respected leader in studies of the treatment of peptic ulcer, gastric cancer, hypertension, endocrine disorders, obesity, and the reversal of atherosclerosis. The famed Lester Dragstedt said that “…the world-wide acceptance of vagotomy is very likely largely due to you and your fine speeches.” (Box 3, Folder 31: Letter to H. William Scott, Jr., MD, from Lester Dragstedt, MD, PhD, October 27, 1970. Personal Communication.). Based on his ongoing animal studies, Dr. Scott enthusiastically said that “…we do believe that it may be possible to reverse existing atherosclerosis by ileal bypass.” (Box 3, Folder 31: Letter H. William Scott, Jr., MD, to Lester Dragstedt, MD, PhD, February 24, 1969).

In addition, he was keenly interested in surgical education and the issues surrounding government control of physicians. Later on, as he aged, he developed yet another interest, that of surgical care for geriatric patients.

The bedside teaching of the medical students and residents was emphasized. He had a penchant for perfectionism and an uncompromising devotion to his profession. This was manifested in his nearly constant work and multiple efforts in patient care, teaching, research, and administration, both at the local institutional level and at the level of multiple national organizations. He was constantly called upon to deliver lectures at multiple institutions. His efforts seemed to bear fruit, however, as is evident from the multiple letters from former trainees in his files. Each of them speaks to the unique and life-enhancing experiences they had as a surgical resident under his tutelage.

Dr. Scott was credited with the creation of an environment that allowed his trainees to grow and to develop skills, knowledge, and attitudes. One trainee said, “I will try to always remember your often proven practice of matching aggressive surgical therapy to aggressive illness, particularly neoplasia. Perhaps of greater day to day importance, I hope to always exhibit in my dealings with patients, their families and other physicians the professional attitudes and actions you are so consistent in demonstrating… I do realize that more than any other individual you were responsible for creating an environment in which I could learn from a number of situations and other surgeons.” (Box 5, Folder 4: Letter to H. William Scott, Jr. from AR Kessler, MD, July 14, 1981. Personal Communication.).

His ideals for training residents were most likely forged from his time as a surgical resident in Boston and later in Baltimore. Unquestionably, his service was difficult due to the pace of activity on it as well as the hours worked. Scott was himself a demanding taskmaster, yet his basic fairness, high ideals, and his superb capabilities as a teacher were instrumental in creating an extremely competitive and highly sought residency. It is said that what he looked for in potential house staff was “an inquiring mind, hallmarks of scholarship, and that the person was naturally dexterous.” (Box 5, Folder 3: Transcript of Interview of H. William Scott, Jr., MD, by Andrew Dale, MD, 1993. Personal Communication.).

## ACCUMULATED HONORS-BRINGING RECOGNITION TO VANDERBILT

**FIGURE 1.**
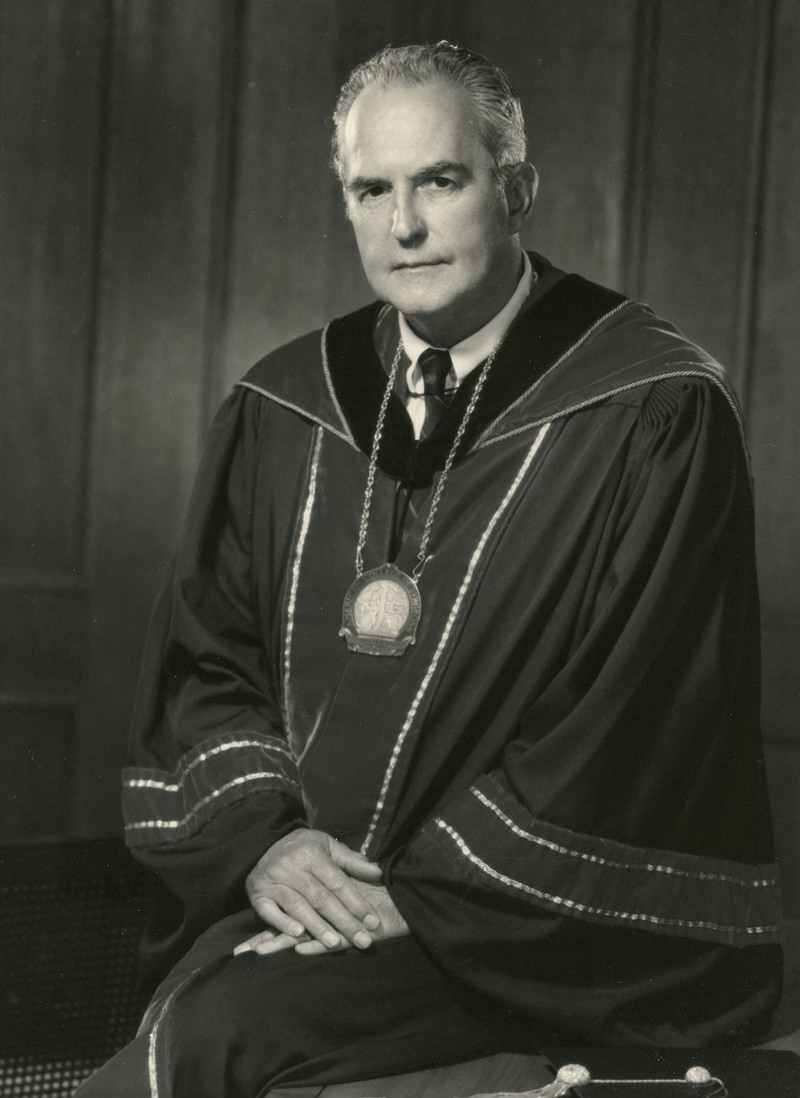
H. William Scott, Jr., MD, 1975.

Dr. Scott accumulated many honors during his time at Vanderbilt. Among these, he was President of the following organizations: Society of University Surgeons (1960–1961), Nashville Surgical Society (1963–1964), Tennessee Chapter American College of Surgeons (1968–1969), Halsted Society (1968–1971), Society for Surgery of the Alimentary Tract (1970–1971), Society of Clinical Surgery (1970–1972), American Surgical Association (1973–1974), American College of Surgeons (1975–1976), (Fig. 1), Southern Surgical Association (1976–1977), Pan Pacific Surgical Association (1980–82), US Chapter International Society of Surgery (1982–83), and International Surgical Group (1982–83). In addition, he was a member of the Board of Regents, American College of Surgeons (1972–1976), a director of the American Board of Surgery (1956–1962), Vice Chair of the American Board of Surgery (1961–1962), and Chair of the NIH Surgery Study Section B (1966–1970).

He was honored by being a Visiting Professor 77 times, was given the degree of Doctor of Science by the University of Aberdeen, and received an honorary degree from the University of Gothenburg. Four societies named him an Honorary Fellow. He was the author or coauthor of more than 350 articles, and he was selected for admission to the Johns Hopkins University Society of Scholars.

As President of the American Surgical Association, Dr. Scott called upon its members to put our professional house in order and to petition the government for a redress of grievances. “We have deep concern that much of the health legislation currently proposed would, if enacted, worsen the delivery of medical and surgical care in America and restrict the professional freedom of American physicians and surgeons.” He was concerned particularly with the efforts of the Nixon administration to shave money out of the National Institutes of Health budget and to impose controls on physicians’ fees and hospital charges. Furthermore, he said that “In the period since World War II there has been a steadily increasing incursion of the federal government into all aspects of health and medical care.”^2^

During his address delivered during the Convocation of the Clinical Congress as President of the American College of Surgeons, Dr. Scott reviewed the major aspects of the professional liability crisis facing many surgeons of the day. He discussed both short- and long-range approaches as possible solutions, and he said that “…hard data on these subjects are very scarce.” He noted, “…the total unavailability of professional liability insurance in some areas of the country plus the exorbitant premium rates proposed by the few remaining insurance carriers…”. Furthermore, he said that “Iatrogenic medical injury presents a uniquely important challenge to the College for investigation and control.” (Scott, HW, Jr. Professional Liability-the crisis and approaches to the solution. *Bull Amer Coll of Surgeons.* 1975; November.)

## TRANSITION IN THE SECTION OF SURGICAL SCIENCES

The new Vanderbilt University Hospital finally opened in September 1980, and in May 1982, Scott was named Professor of Surgery Emeritus. (Scott, HW, Jr. 1981-1982. In: Scott, Jr. HW, Herrington JL, Rosenfeld L, Sawyers JL, eds. History of Surgery at Vanderbilt. Nashville; 1996: 291-300.) He retired from his chairmanship on June 30, 1982, after holding that post for 30 years. He said, “I know I will go down the tubes if I don’t stay active.” He did not have to worry about that for long because he was asked to stay on as interim Chair until such time as his successor, Dr. John Cameron, could come from Johns Hopkins and take up the post. Cameron was expected to arrive in early August, but he elected to remain in Baltimore. Dr. Scott became energized in support of his first choice as a possible successor, Dr. John Sawyers, who was soon named the interim head of the Section of Surgical Sciences. Scott’s efforts to help Dr. Sawyers secure the appointment were ultimately successful, as Sawyers took the reins of the section on a permanent basis early in 1983.

Before this time there had never been a funded chair in surgery. This all changed upon Dr. Scott’s retirement as Chair, as he related to a professional colleague, “Last week Mary and I were given a dinner by Vice Chancellor Robinson and his wife with a large group of our faculty and close friends present. At that time they announced the funding of a Chair of Surgery at Vanderbilt in my honor. Naturally, I was overwhelmed.” (Box 7, Folder 4: Letter H. William Scott, Jr., MD, to Frank Spencer, MD, October 11, 1982. Personal Communication.). This was followed closely by the establishment of another chair, the John C. Foshee Distinguished Chair of Surgery.

## ADDITIONAL CONSIDERATIONS

Dr. Scott rose to prominence at an interesting time in history. There were increasing calls for surgery, and all of medicine, to be placed on a firm scientific basis. The early dramatic improvements in this regard served only to fuel the expectations of future advances and improved treatments of diseases and conditions. (Jacobson, TC. Making Medical Doctors: Science and Medicine at Vanderbilt Since Flexner. The University of Alabama Press, Tuscaloosa, AL; 1987:1–349). He dealt with these expectations through an intensive program of laboratory investigations, which led to further knowledge and led to enhanced training for his residents who spent additional time in the laboratory. Many of the current interests of his day and the understanding of disease processes at that time were noted by his publications and presentations having to do with vascular and open-heart surgery, surgery for peptic ulcer disease, transplantation, surgical treatment of obesity, surgical nutrition, surgery of the aged, and advances in surgical education. (Sparkman, RS, ed. The Southern Surgical Association. The First 100 Years. 1887-1987. Philadelphia; 1989: 1-533.) His example of assuming multiple national and international leadership positions was exemplary.

Dr. Scott was certainly very tough and demanding, especially when it came to dealing with his house staff on a day-to-day basis. This was readily apparent. What was not so apparent were his dealings with members of his staff and others. His files are replete with letters that he sent to former residents, faculty associates, and others that together reveal the softer aspects of his character. One example is the following: “I am much interested in his welfare and hope that he will be able to secure a practice relationship which will get him back on his feet again and ultimately bring him the fulfillment which he deserves.” (Box 2, Folder 26: Letter H. William Scott, Jr., MD, to Burneice Larson, May 11, 1960. Personal Communication.). In another letter he offered his sympathy, “I want to offer you my heartfelt sympathy in your bereavement and tell you how terribly, terribly sorry I am about this tragedy.” (Box 5, Folder 4: Letter H. William Scott, Jr., MD, to Mrs. Ralph Larsen, May 26, 1964. Personal Communication.).

He was a very strong proponent of professionalism in all its aspects, including the responsibilities of a surgeon. Evidence for this can be found in the following statement made after an inquiry about rendering an opinion in a legal matter, “I am afraid that I feel rather strongly that the operating surgeon has the responsibility to review the pathologic diagnosis and follow that patient himself in the postoperative period.” (Box 3, Folder 31: Letter H. William Scott, Jr., MD, to Marvin Deck, Jr., MD, February 11, 1975. Personal Communication.). He also had strong opinions about the ethics of surgeons, revealed in the following, “I will never be able to accept the concept that a member of the medical profession behaves ethically and appropriately in advertising his skills.” (Box 5, Folder 4: Letter H. William Scott, Jr., MD, to Terry Lilly, Jr., MD, September 12, 1983. Personal Communication.).

## TOWARDS THE END

Dr. Scott remained an active clinician and teacher and writer for many years following his transition to Professor Emeritus. He maintained his surgical practice and taught medical students and residents through active bedside teaching rounds, and he was still frequently called upon as a visiting professor. He continued to write extensively, and he was the editor or coeditor of 4 textbooks, including Surgical Care of the Elderly, Surgery of the Adrenal Glands, Surgery of the Stomach, Duodenum, and Small Intestine, and History of Surgery at Vanderbilt University. He would say of this time, “I suppose I am a nut for keeping on after a reasonable retirement age, but somehow, I haven’t found time to retire, and nobody has led me away as yet to tell me I should, so I just haven’t.” (Box 4, Folder 5: Letter H. William Scott, Jr., MD, to David Hall, DMD, MD, February 19, 1990. Personal Communication.).

He kept up a lively correspondence, especially with his former trainees, many of whom made him especially proud. “I now have 27 of these framed pictures on the wall in my office representing our Surgical Residents who have become full Professors, and I am so proud that the number has recently gone up to 30. I look forward to adding your picture to the group.” (Box 7, Folder 11: Letter H. William Scott, Jr., MD, to Eugene Woltering, MD, May 4, 1994. Personal Communication.). Words of praise and gratitude for having served as such a good example during their training continued to pour in from former surgery residents. “Your example of being not only a superb surgeon but a true gentleman are lessons that I will carry with me always.” (Box 1, Folder 17: Letter to H. William Scott, Jr., MD, from Daniel Benckart, MD, June 29, 1982. Personal Communication.). Also, “Your emphasis on clinical acumen, intellectual objectivity, and meticulous operative technique have formed the axis of my surgical training, but I particularly want to thank you for the example which you have set for me with regard to courtesy, neatness, and promptness…I have become a student of the high standards that govern the way you practice medicine, and I have become less amazed by good results”. (Box 5, Folder 4: Letter to H. William Scott, Jr., MD, from Robert Kieffer, MD, June 23, 1984. Personal Communication.).

## FINAL THOUGHTS

Dr. Scott died on August 5, 1998, and was buried in Linwood Cemetery in his hometown of Graham, North Carolina. He was truly a force to be reckoned with, and he left behind a tremendous legacy of surgical care, investigation, teaching, departmental administration, and leadership.

He was, above all, a demanding task master, as demonstrated in the following letter from his files. “Those of us fortunate enough to be your students received far more than an understanding of the basic principles of surgery. For an astute few, you gave us an appreciation of the history and nobility of our profession.…I’m sure history will regard you, along with these men (Cutler, Blalock, and Brooks), as a major contributor to modern surgical practice. Throughout my career, I will remember the standards that you demand, and attempt to attain them.” (Box 4, Folder 1: Letter to H. William Scott, Jr., MD, from James Givens, MD, July 10, 1977. Personal Communication.).

There is one more letter that speaks to his insistence on high standards and possibly best summarizes all that he did and meant to Vanderbilt. “I also feel very strongly that the total impact of your example, training, and insistence went well beyond what any of us at Vanderbilt and Hopkins would have normally expected. Small details of concern for the patient, such as the insistence that old men with head and neck lesions would be shaved for your rounds, even if it meant that the house officer did the shaving, carried with it a concern for the patient, for the high standards of the art and craft and science of surgery that were unique to Vanderbilt.” (Box 43, Letters, H. William Scott, Jr., MD 1923–1997, Letter to H. William Scott, Jr., MD, from Ide Smith, MD, February 15, 1989. Personal Communication.).

May his legacy be remembered and celebrated, and may it be perpetuated.

## ACKNOWLEDGMENTS

The authors wish to acknowledge the very capable assistance of Christopher Ryland, Curator of the History of Medicine Collection and Archives and James Thweatt, Archivist, of the History of Medicine Collections of the Eskind Biomedical Library of Vanderbilt University. We also thank Marjorie Tattersfield, Assistant to the Chair, Section of Surgical Sciences, for her invaluable assistance with editing. Unless otherwise noted, all references pertain to correspondence from the files of the H. William Scott, Jr. Collection of the Annette and Irwin Eskind Biomedical Library Archives, Vanderbilt University.
